# Supplementary material for: Cryo-EM reveals the molecular basis of laminin polymerization and LN-lamininopathies
Source: Nat Commun. 2023 Jan 19;14:317. doi: 10.1038/s41467-023-36077-z (PMC9852560; doi:10.1038/s41467-023-36077-z)
Supplement: Supplementary file 1 — Supplementary Information [file 41467_2023_36077_MOESM1_ESM.pdf]

Cryo-EM reveals the molecular basis of laminin polymerization and LN-lamininopathies.

Arkadiusz W. Kulczyk[1*], Karen K. McKee[2], Ximo Zhang[3], Iwona Bizukojc[3,4], Ying Q. Yu[3] & Peter D. Yurchenco[2]

[1]Institute for Quantitative Biomedicine, Department of Biochemistry and Microbiology, Rutgers University, Piscataway, NJ 08854, USA
[2]Department of Pathology and Laboratory Medicine, Rutgers - Robert Wood Johnson Medical School, Piscataway, NJ 08854, USA
[3]Waters Corporation, Milford, MA 01757, USA
[4]Cryo-EMcorp, Bridgewater, NJ 08807, USA

[*]Correspondence should be addressed to Arek Kulczyk (arek.kulczyk@rutgers.edu)

Inventory of Supporting Information:

**Supplementary Table 1**	Cryo-EM data collection, refinement and validation statistics.
**Supplementary Table 2**	The Q-score calculations confirm the assignment of individual Lm subunits in the map of the Lm polymer node.
**Supplementary Figure 1**	Cryo-EM structure determination of Lm polymer node.
**Supplementary Figure 2**	Structural features of the Lm polymer node.
**Supplementary Figure 3**	The SPA workflow employed for cryo-EM structure calculations.
**Supplementary Figure 4**	Effects of the directional resolution anisotropy on structural reconstructions of the Lm polymer node.
**Supplementary Figure 5**	Validation of the molecular model calculated for the trimeric Lm polymer node.
**Supplementary Figure 6**	Calculations of the initial model of the Lm polymer node using a program AlphaFold2.
**Supplementary Figure 7**	The individual Lm subunits adopt distinct conformations within the trimeric Lm polymer node, and exhibit unique N-glycosylation footprints.
**Supplementary Figure 8**	A superposition of maps and models representing individual Lm subunits
**Supplementary Figure 9**	The MS glycopeptide analysis of the Lm polymer node.
**Supplementary Figure 10**	Dynamic behavior of the Lm polymer node.
**Supplementary Figure 11**	Interactions stabilizing a structure of Lm polymer node.
**Supplementary Figure 12**	Interactions stabilizing the Lm polymer node.
**Supplementary Figure 13**	Structural and functional analysis of the inter-subunit interfaces formed by β1 with γ1.
**Supplementary Figure 14**	The mutagenesis analysis of the trimeric Lm polymer node.
**Supplementary Figure 15**	Structural analysis of the inter-subunit interfaces formed by α1 with β1 and γ1 explains mechanistic basis for the amino-acid substitutions disrupting the trimeric structure of the Lm polymer node.
**Supplementary Figure 16**	Structural and functional analysis of the inter-subunit interfaces formed by β1 with α1 and γ1.
**Supplementary Figure 17**	Structural and functional analysis of the inter-subunit interfaces formed by γ1 with α1 and β1.
**Supplementary Figure 18**	Structural details of the α1-γ1 interface.
**Supplementary Figure 19**	Sequence alignment of Lm β1 and Lm β2.
**Supplementary References**	

Supplementary Figures and Table

	Lm polymer node (EMDB-27542 [https://www.ebi.ac.uk/emdb/EMD-27542]) (PDB 8DMK [https://www.wwpdb.org/pdb?id=pdb_00008dmk])
**Data collection and processing**	
Magnification	130,000x
Voltage (kV)	300
Electron exposure (e–/Å^2)	52.3
Defocus range (μm)	0.8-2.2
Pixel size (Å)	0.324
Symmetry imposed	C1
Initial particle images (no.)	1,068,172
Final particle images (no.)	125,011
Map resolution (Å)	3.7
FSC threshold	0.143
Map resolution range (Å)	3.4-5.6
**Refinement**	
Initial model used (PDB code)	N/A
Model resolution (Å)	N/A
FSC threshold	N/A
Model resolution range (Å)	N/A
Map sharpening *B* factor (Å^2)	-100.8
Model composition	
Non-hydrogen atoms	7571
Protein residues	946
Ligands	6
B factors (Å^2)	
Protein	89.41
Ligand	50.14
R.m.s. deviations	
Bond lengths (Å)	0.002
Bond angles (°)	0.527
Validation	
MolProbity score	2.00
Clashscore	11.9
Poor rotamers (%)	3.00
Ramachandran plot	
Favored (%)	95
Allowed (%)	5
Disallowed (%)	0

Supplementary Table 1 Cryo-EM data collection, refinement and validation statistics.

	Assigned Segment in the cryo-EM map		
	Lm α1	Lm β1	Lm γ1
**Atomic model**			
Lm α1	**0.42 (3.9 Å)**	0.17	0.19
Lm β1	0.16	**0.4 (4.1 Å)**	0.16
Lm γ1	0.2	0.17	**0.44 (3.8 Å)**

Supplementary Table 2 The Q-score calculations confirm the assignment of individual Lm subunits in the map of the Lm polymer node. The Q-score values indicate the resolvability of individual atoms in a map and reflect the agreement of the model with the map[1]. We have employed the backbone Q-score calculations to investigate the correctness of Lm subunit assignment in the cryo-EM map of the trimeric Lm polymer node. The Q-scores were calculated for each possible model-map combination. Only one unique model-map pair for each α1, β1, and γ1 has substantially higher Q-score values (highlighted in green in the table) than other pairs, confirming an unambiguously assignment of Lm subunits to the map. The Q-score values calculated for these unique map-model pairs are consistent with the Q-score values expected for a map at the reported resolution.

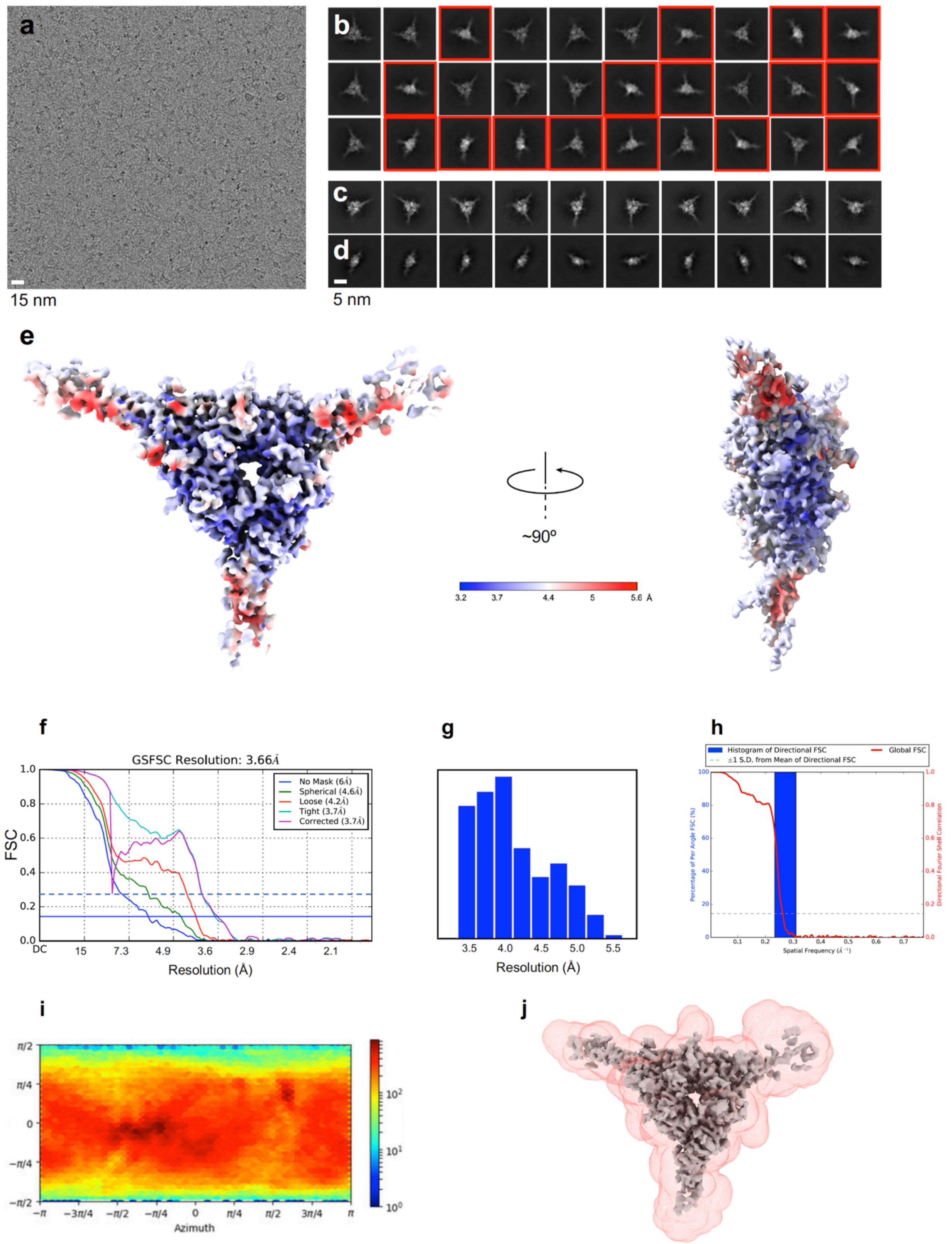
a
b
c
d
15 nm
5 nm
e
~90º
3.2
3.7
4.4
5
5.6 Å
f
g
h
GSFSC Resolution: 3.66Å
No Mask (6Å)
Spherical (4.6Å)
Loose (4.2Å)
Tight (3.7Å)
Corrected (3.7Å)
FSC
Resolution (Å)
Histogram of Directional FSC
Global FSC
±1 S.D. from Mean of Directional FSC
i
j
Azimuth

Supplementary Figure 1 Cryo-EM structure determination of Lm polymer node. (**a**) One of 37,927 representative averaged micrographs used for particle picking. The image shows approximately 200-300 particles dispersed across the field of view. Micrographs were acquired with a Titan Krios electron microscope equipped with the K3 direct electron detector. (**b**) Representative 2D class averages of Lm trimers obtained at the protein concentration of 90 μg/ml. The 2D classes display both front/rear and side/tilted views (red squares) of Lm polymer node. (**c**) Representative 2D classes of Lm polymer node at protein concentration of 60 μg/ml. The majority of classes represent front and rear views of the complex. (**d**) At 120 μg/ml, the majority of 2D classes present the side views of the complex. (**e**) Local resolution values calculated in the program DeepRes[2] mapped onto the surface of Lm polymer node structure. (**f**) Resolution of the Lm polymer node structure calculated using the "gold standard" Fourier Shell Correlation (FSC) method according to the 0.143 criterion in cryoSPARC v3 is 3.66 Å. (**g**) Local resolution histogram calculated using the program DeepRes[2]. (**h**) The angular distribution plot confirms the complete and nearly uniform coverage of the angular space. (**i**) The directional FSC plot confirms isotropic sampling of Fourier components in the structure of Lm polymer node. (**j**) The mask applied in cryoSPARC v3 was not too tight, and it encompassed all parts of the 3D map. Source data are provided as a Source Data file.

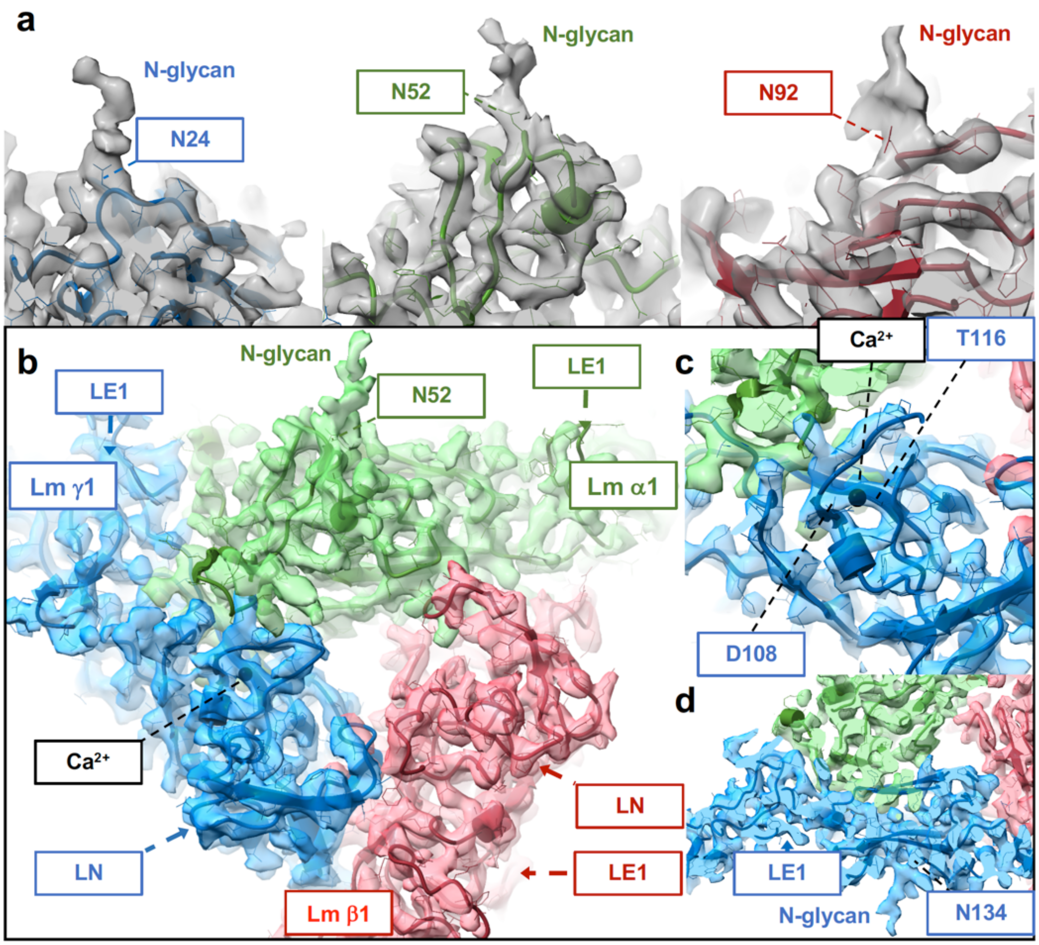

Supplementary Figure 2 Structural features of the Lm polymer node. (**a**) EM densities representing N-glycans in the map. Lm subunits are color-coded: α1, β1 and γ1 are shown in green, red and blue, respectively. (**b**) The top view on the inter-subunit interfaces within Lm trimer. A calcium ion is displayed as a black sphere. (**c**) Calcium-binning side in γ1 with labeled metal-coordinating residues. (**d**) A side view of the LE1 domain in γ1.

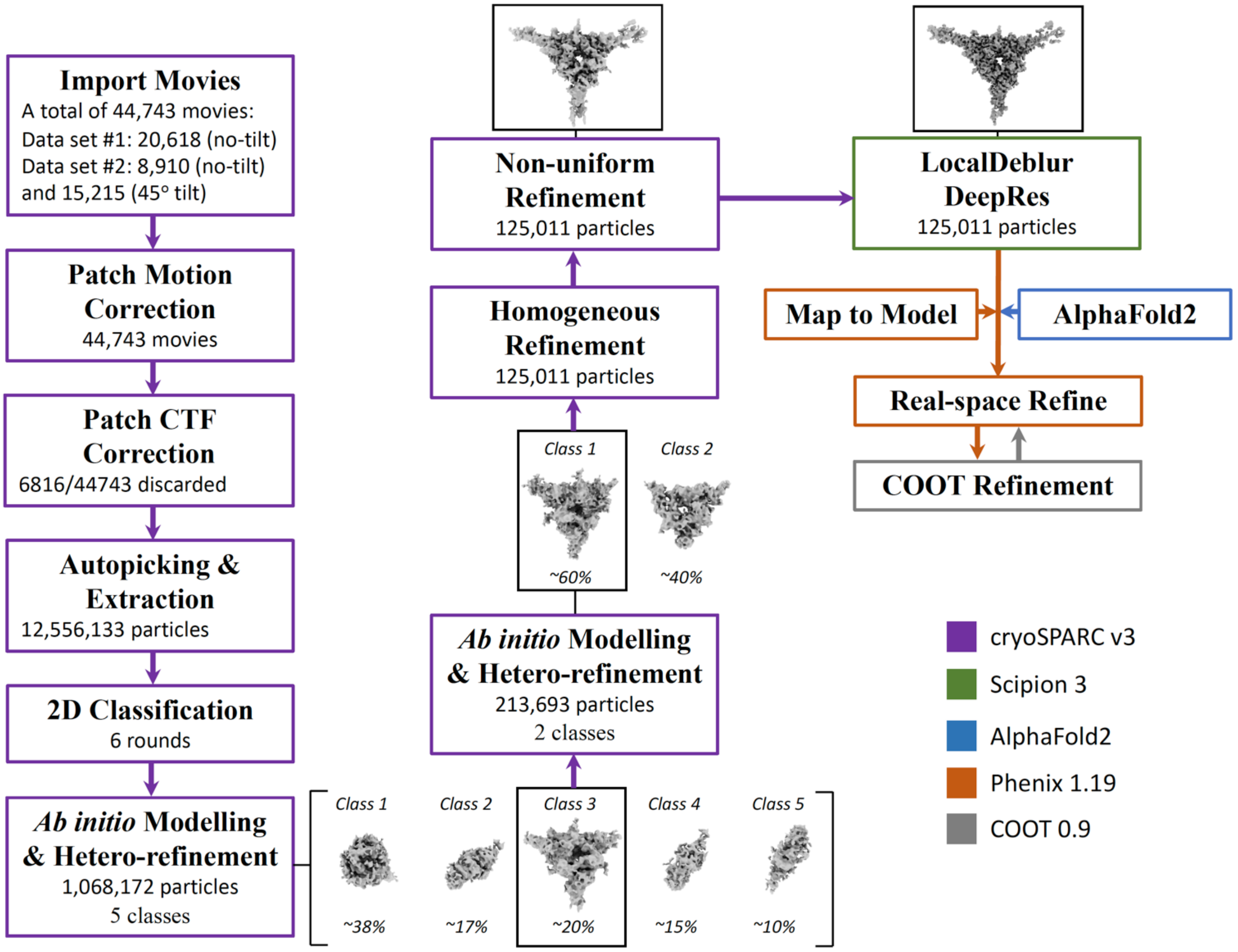

Supplementary Figure 3 The SPA workflow employed for cryo-EM structure calculations. We employed a combination of cryoSPARC v3, Scipion 3, AlphaFold2, Phenix 1.18 and COOT 0.9 for image processing and structure calculations of the Lm polymer node. Steps completed in the above-mentioned programs are denoted in purple, green, blue, red and gray boxes, respectively. Please see sections concerned with structure calculations and model building in Methods for details.

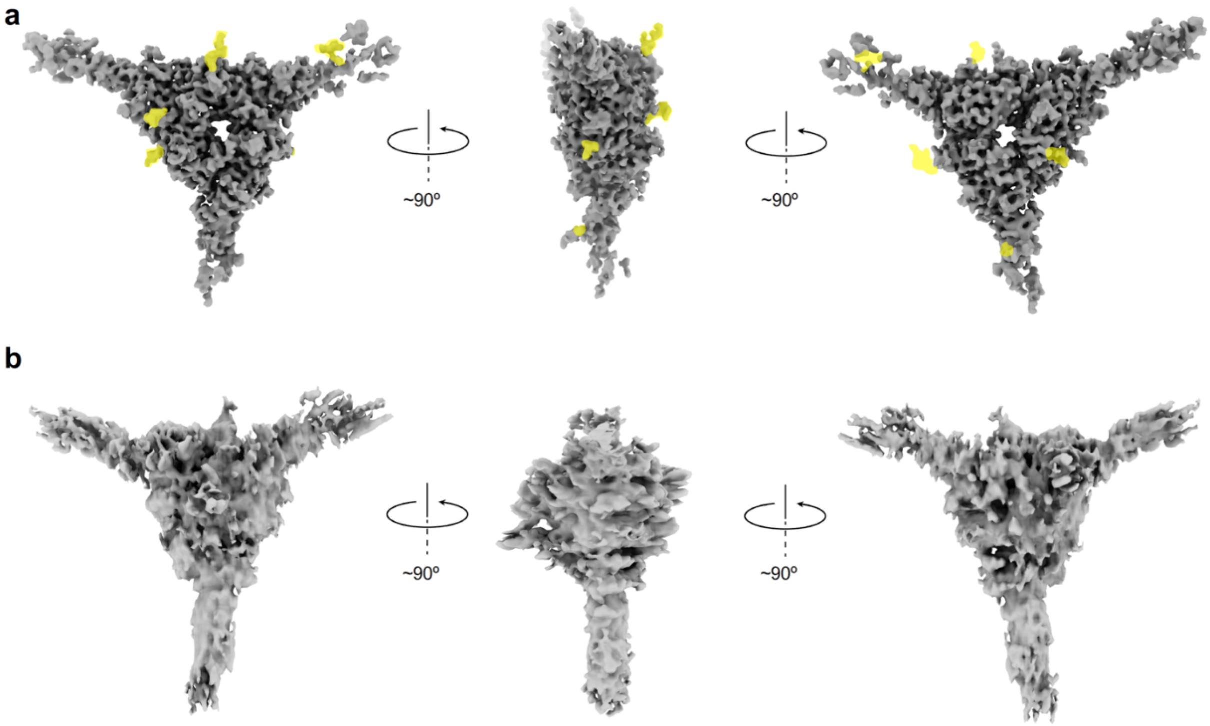

Supplementary Figure 4 Effects of the directional resolution anisotropy on structural reconstructions of the Lm polymer node. A comparison of 3D reconstructions obtained with either (**a**) a mixture of movies acquired at no stage tilt and at 45^0 tilt, or (**b**) by using only the movies collected at no stage tilt. The latter reconstruction clearly exhibits the effects of under-sampling of Fourier components and the directional resolution anisotropy parallel to the preferred orientation axis, which is particularly pronounced in the side view of the complex. The N-glycans identified in the isotropic map are colored in yellow.

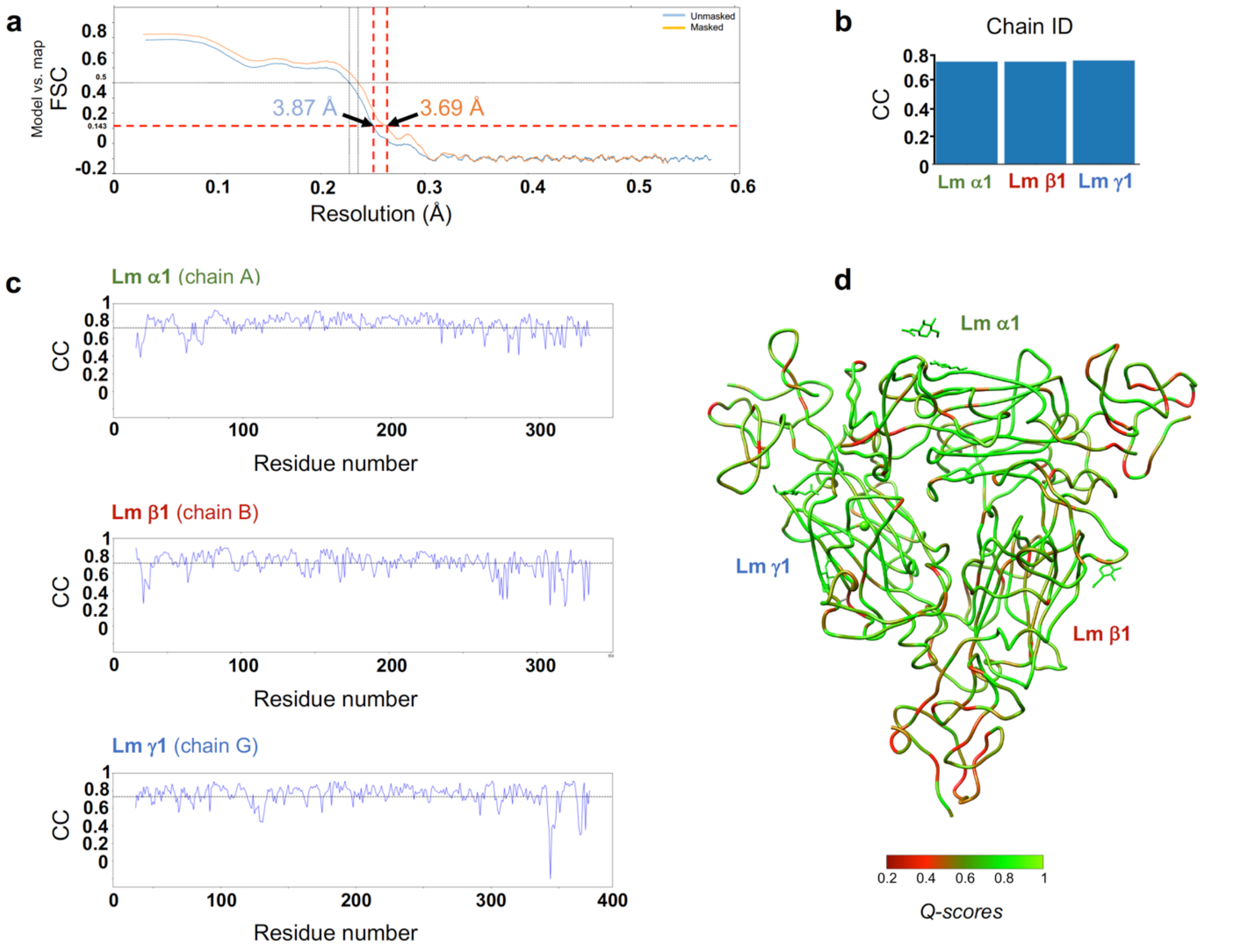

Supplementary Figure 5 Validation of the molecular model calculated for the trimeric Lm polymer node. (**a**) The output from phenix.mtriage. The graph shows FSC curves representing the model vs. map cross-correlation with and without masking. The model displays a good agreement with the map, with the gold-standard FSC values at 0.143 of 3.69 Å and 3.87 Å, calculated for the masked and unmasked maps, respectively. These values are consistent with the reported resolution of 3.7 Å (**Supplementary Figure 1f-h**). (**a**) The output from phenix.validation_cryoem showing cross-correlation of individual protein chains and the map. (**c**) The output from phenix.validation_cryoem displaying cross-correlation coefficients calculated for the model vs. map per residue for each individual chain. (**d**) The Q-score values calculated with MapQ[1] confirm the agreement of the model with the map. Lower Q-score values at the C-termini of α1, β1, and γ1 reflect the intrinsic dynamics of the LE domains, as revealed by the Principal Components Analysis. Source data are provided as a Source Data file.

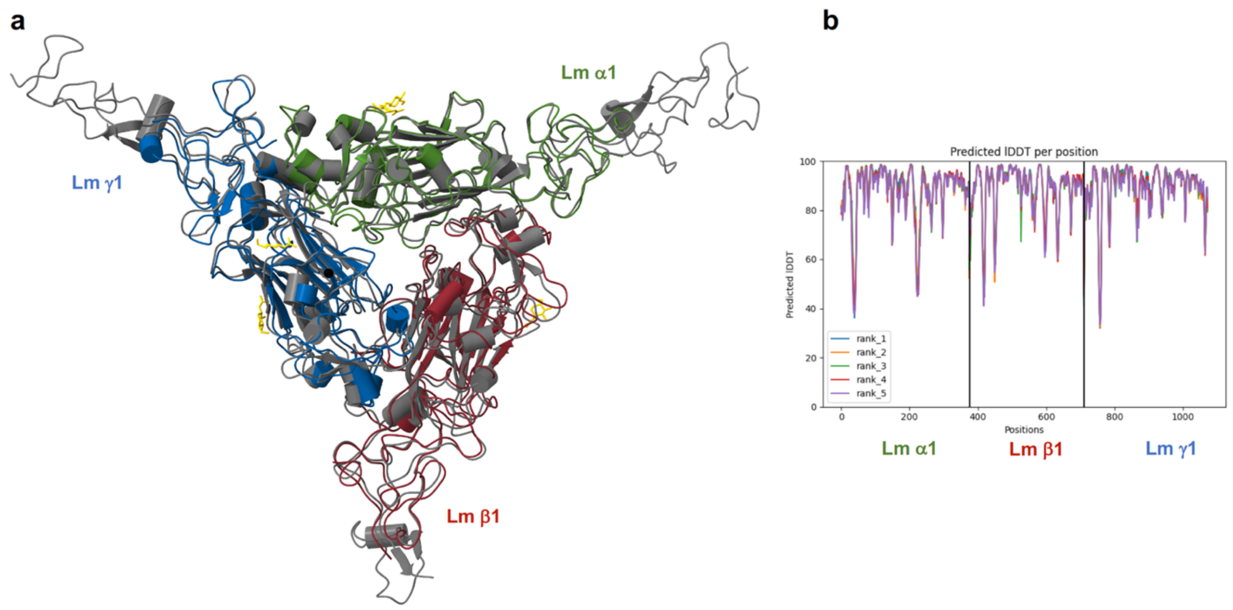

Supplementary Figure 6 Calculations of the initial model of the Lm polymer node using a program AlphaFold2[3]. (**a**) A superposition of the model obtained with AlphaFold2 (gray) and the final molecular model of the Lm polymer node. Individual Lm chains are color-coded in the final model, namely α1 in green, β1 in red, and γ1 in blue. The backbone RMSD across 305 atom pairs is 2.41 Å. (**b**) Predicted Local Distance Difference Test (lDDT) values per each amino acid in the Lm polymer node sequence. The initial AlphaFold2 model was calculated with a total of 1072 amino acids corresponding to the sequences of α1, β1 and γ1 subunits.

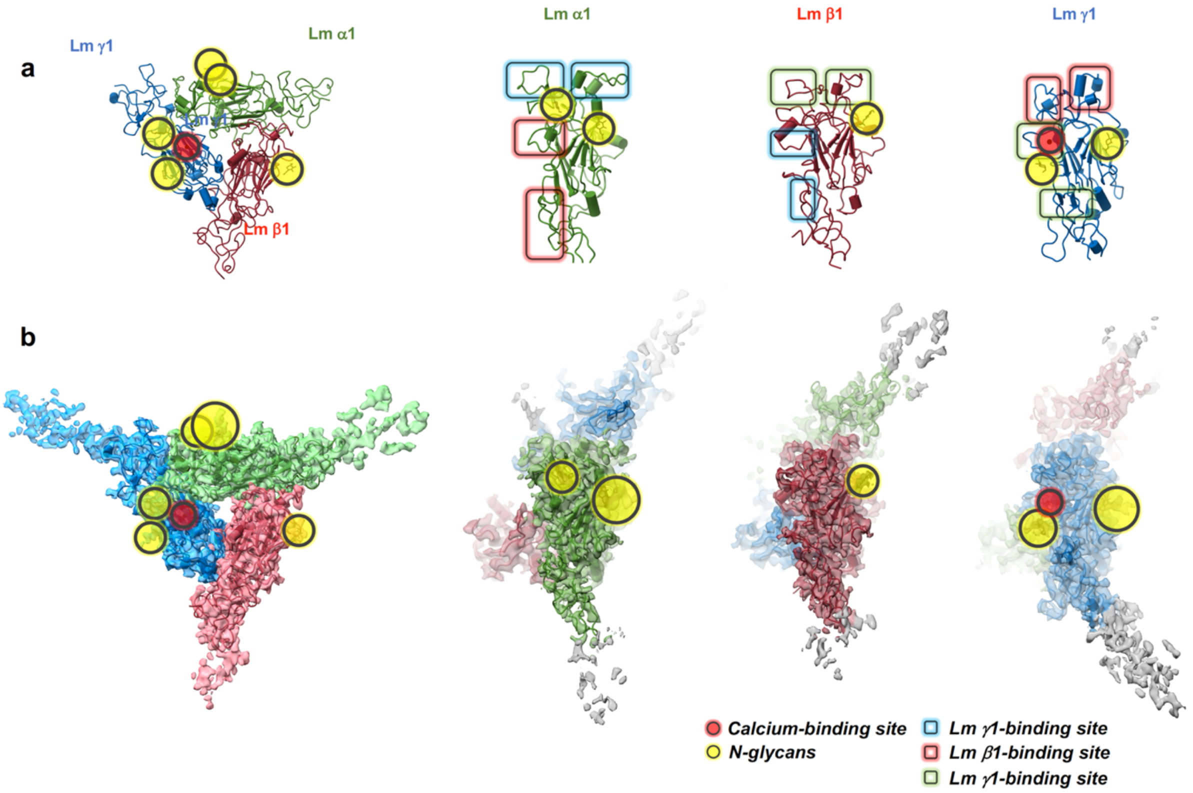

Supplementary Figure 7 The individual Lm subunits: α1 (green), β1 (red) and γ1(blue) adopt distinct conformations within the trimeric Lm polymer node, and exhibit unique N-glycosylation footprints. (**a**) Atomic models of the trimeric complex along with the models of individual subunits. The unique inter-subunit interfaces and the N-glycosylation sites are mapped onto the models. The α1-binding sites in β1 and γ1 are denoted by green boxes. The β1-binding sites in α1 and γ1 are shown as red boxes. The γ1-binding sites in α1 and in β1 are displayed as blue boxes. As shown, the loops involved in the inter-subunit interactions from α1, β1 and γ1 are substantially different in length and in the conformations they adopt to form a trimeric complex. The distinctive N-glycosylation sites are indicted by yellow circles. Additionally, the calcium coordination site could be identified in γ1, and it is shown in the red circle. (**b**) Atomic models superimposed with the cryo-EM Coulomb map of the Lm polymer node in analogous orientations with inter-subunit interfaces, N-glycan- and calcium-binding sites denoted as above.

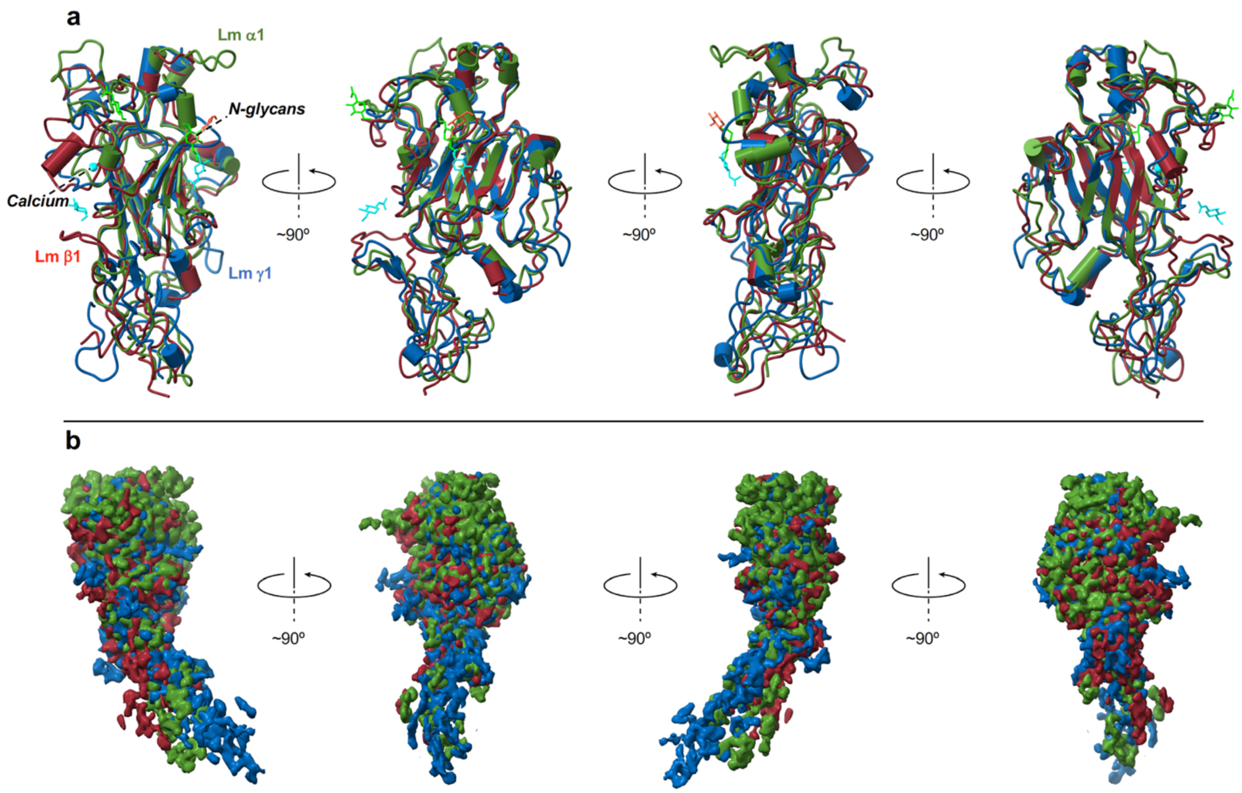

Supplementary Figure 8 A superposition of maps and models representing individual Lm subunits. A superposition of **(a)** atomic models of α1 (green), β1 (red) and γ1 (blue), and **(b)** segmented map fragments corresponding to individual Lm subunits. For clarity, the ligands (N-glycans and a calcium ion) are colored like the maps and models they are associated with. The LN domains of α1, β1, and γ1 subunits contain conserved seven- or eight-stranded antiparallel β-sheets, which superimpose with the backbone RMSDs of: 1.23 Å (over 145 pruned atom pairs from α1 and β1), 1.12 Å (166 pruned atom pairs from α1 and γ1), and 1.09 Å (135 pruned atom pairs from β1 and γ1). However, the remaining parts of the protein subunits are structurally distant with RMSDs of: 4.94 Å (α1 vs. β1), 3.27 Å (α1 vs. γ1), and 5.19 Å (β1 vs. γ1) across all atoms. The cryo-EM maps are substantially different with correlation coefficients of 0.685 (α1 vs. γ1), 0.672 (α1 vs. γ1), and 0.675 (β1 vs. γ1).

a

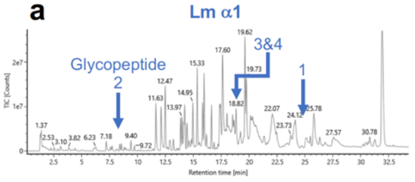

Glycosylation site	Glycopeptide	Identified glycoform				
		M9	F+4HexNAC+S	M8	2F+4HexNAC	M10
1 (N45)	GLFPAILNLATNAHISANATCGEK	2.2%	88.1%	0.4%	8.9%	0.4%
2 (N79)	VNSTNPR		100%			
3 & 4 (N370 & N374)	SLNTAGQYSGGGVCVNCSQNTT GINCETCIDQYYR		100%*		100%*	

b

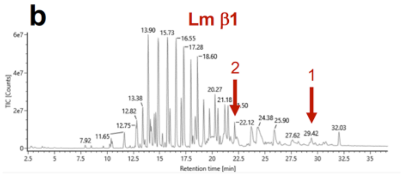

Glycosylation site	Glycopeptide	Identified glycoform			
		FA2BG2S1	A3F2G2	FA2G2S1	FA3G2
1 (N120)	CMPEFVNAAFNVTVVATNTCGT PPEEYCVQTGVTGVTK	21.2%	43.3%	5.9%	29.6%
2 (N356)	CNCNEHSISCHFDMAVYLATGNV SGGVCDDCQHNTMGR		20%		80%

c

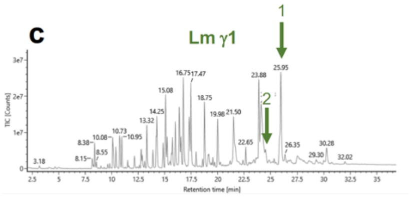

Glycosylation site	Glycopeptide	Identified glycoform				
		FA2G1Sg1	FA2BG2S1	FA2G2S2	A2G2S1	FA2G2S1
1 (N60)	CMPEFVNAAFNVTVVATNTCGT PPEEYCVQTGVTGVTK	6.7%	38.5%	28.8%	6.7%	19.3%
2 (N134)	SCHLCDAGQPHLQHGAAFLTDY NNQADTTWWQSQTMLAGVQY PSSINLTLHLGK	49.5%			49.5%	1%

Supplementary Figure 9 The MS glycopeptide analysis of the Lm polymer node. The glycopeptide mapping revealed eight unique N-glycosylation sites: (**a**) four in α1 (N45, N79, N370, N374), (**b**) two in β1 (N120, N356), and (**c**) two in γ1 (N60, N134). The arrows indicate the observed chromatographic peaks of the peptides bearing one or two glycosylation sites. The released glycan analysis performed for each individual subunit revealed a mixture of approximately 80 glycans. All glycans with the abundance higher than 5% are listed in the tables adjacent to the chromatograms. The listed glycans constitute approximately 49%, 46% and 46% of all glycans identified in the released glycan analysis of α1, β1 and γ1 subunits, respectively. The relative occupancies of the most abundant glycans at each specific N-glycosylation site are summarized in the tables. Nomenclature of monosaccharides: A-antennary; B-bisecting; F-fucose; G-galactose; HexNAC-acetylhexosamine, M-mannose, S-sialic acid (N-Acetylneuraminic acid); Sg-sialic acid (N-Glycolylneuraminic acid). *The analysis of α1 revealed that one of the identified glycopeptides carries two N-glycosylation sites with N370 and N374, each linked to F+4HexNAC+S or 2F+4HexNAC.

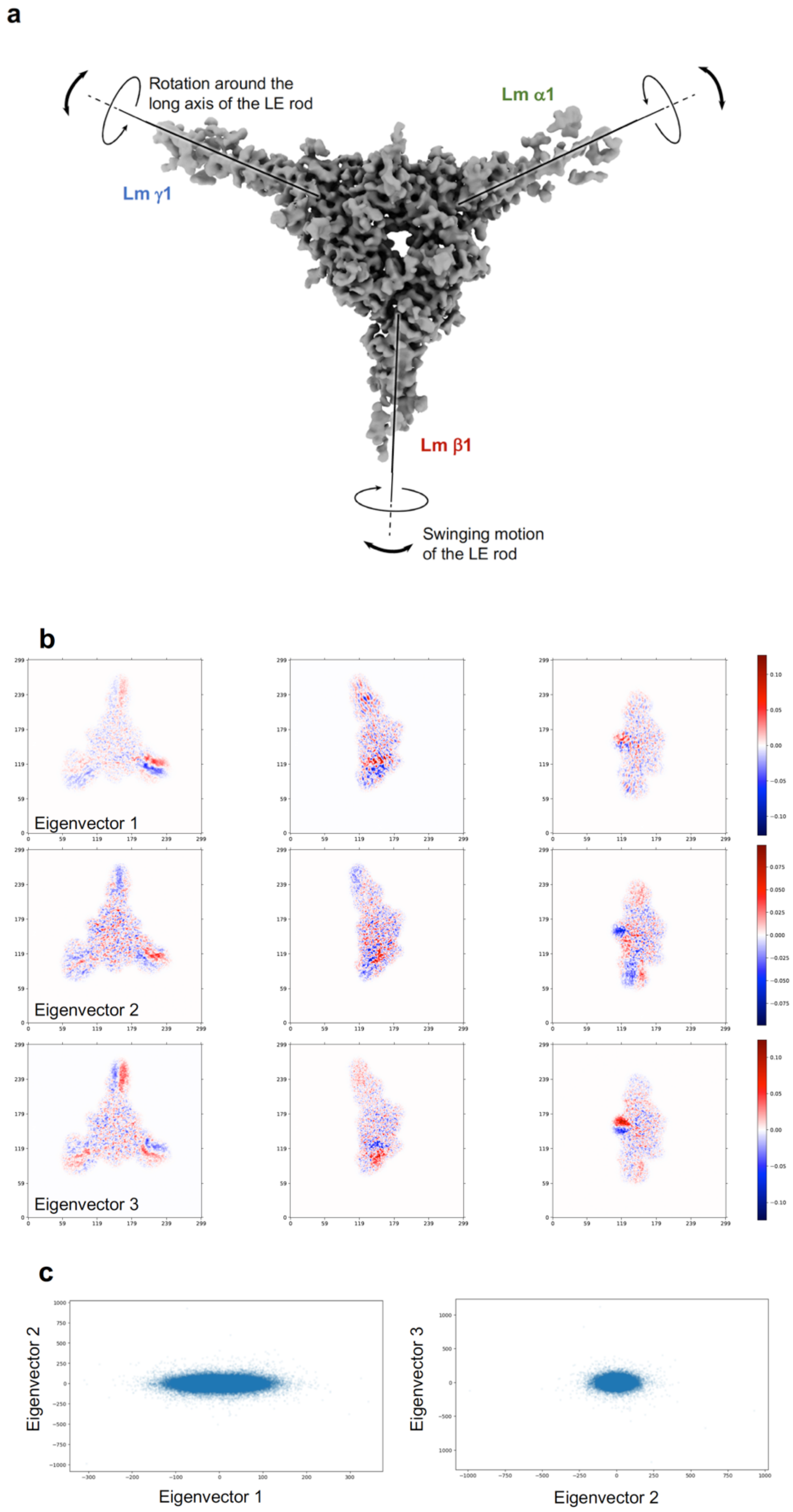
a
Rotation around the
long axis of the LE rod
Lm α1
Lm γ1
Lm β1
Swinging motion
of the LE rod
b
Eigenvector 1
Eigenvector 2
Eigenvector 3
c
Eigenvector 2
Eigenvector 1
Eigenvector 3
Eigenvector 2

Supplementary Figure 10 Dynamic behavior of the Lm polymer node. (a) The Principal Components Analysis (PCA) performed in cryoSPARC v3 revealed the rotational mobility of the LE rods along their long axes, and the planar swinging of the rods along the axes perpendicular to these long axes. Directions of both motions are indicated in the figure by black arrows. **(b)** Three eigenvectors representing variability in the data-set of 213,694 particle images. The variability is characterized by the range of positive (red) and negative (blue) values at each voxel of the volume. Three different projections of each eigenvector volume are shown. **(c)** Reaction coordinates calculated for the set of 213,693 particle images. The distribution of reaction coordinates across particles in the dataset provides information about the variability landscape of the complex by highlighting the populations of particles adopting a specific position in the conformational space spanned by the eigenvectors. These plots illustrate the mobility of the Lm polymer node. The continuous distributions confirm the rotation and swinging-like motions in the complex, rather than the presence of discrete conformational states.

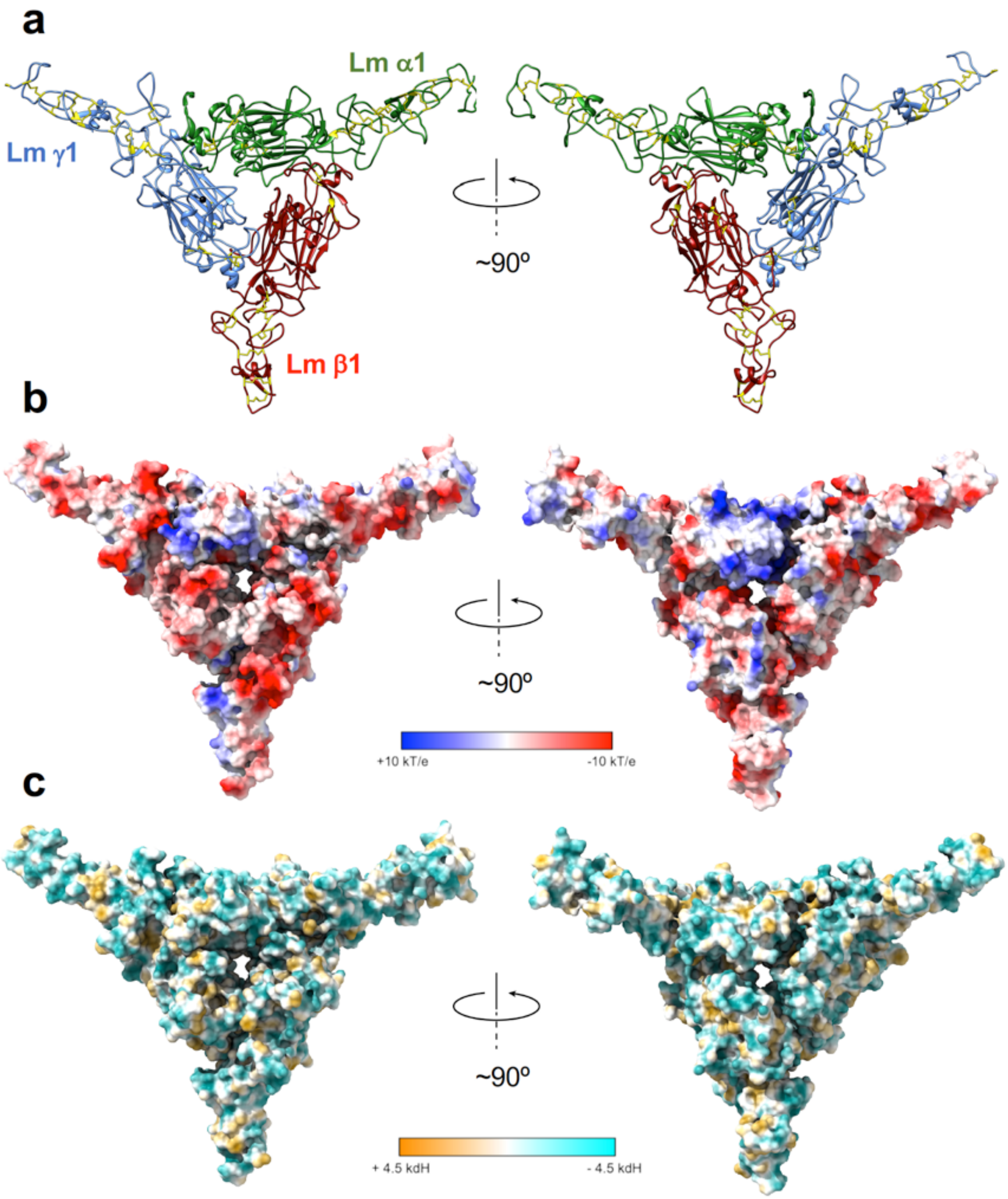

Supplementary Figure 11 Interactions stabilizing a structure of Lm polymer node. (**a**) A network of disulfide bridges is displayed in yellow. There are twelve disulfide bonds in Lm α1, ten in Lm β1 and twelve in Lm γ1 subunits. (**b**) Surface of Lm polymer node was colored according to its electrostatic potential. (**c**) Protein surfaces colored according to the hydrophobicity scale of Kyte and Doolittle.

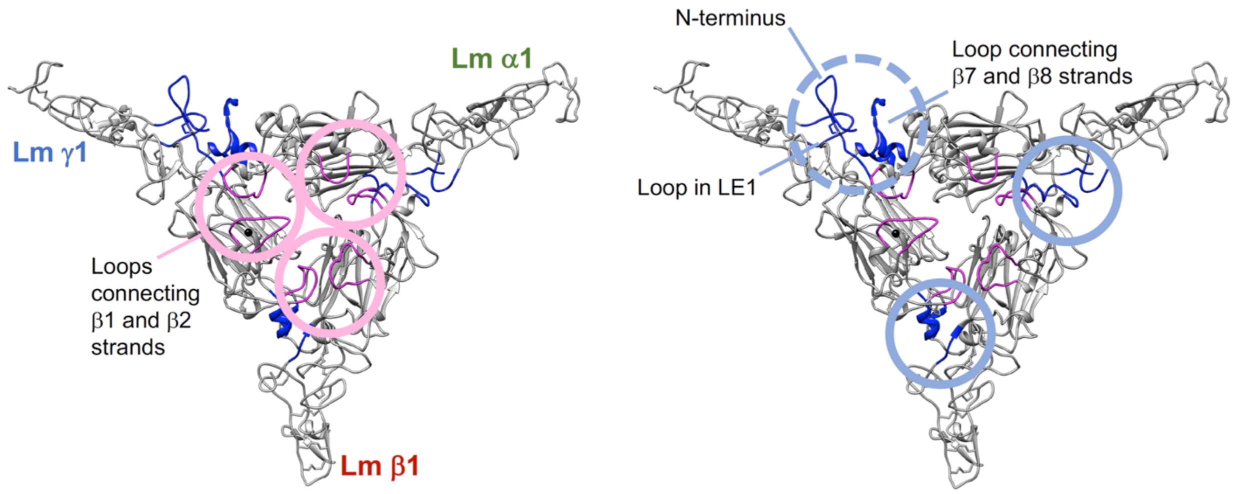

Supplementary Figure 12 Interactions stabilizing the Lm polymer node. A structure of Lm node resembles two triangles, each representing a network of unique inter-subunit interactions. The first group involves loops connecting β1 and β2 strands in β-sheets from neighboring subunits within the trimeric complex. These regions are highlighted in pink. The second set of interactions highlighted in blue includes loops linking strands β7 and β8 from one subunit and the N-terminal regions along with one of the loops from the LE1 domain in the neighboring subunit.

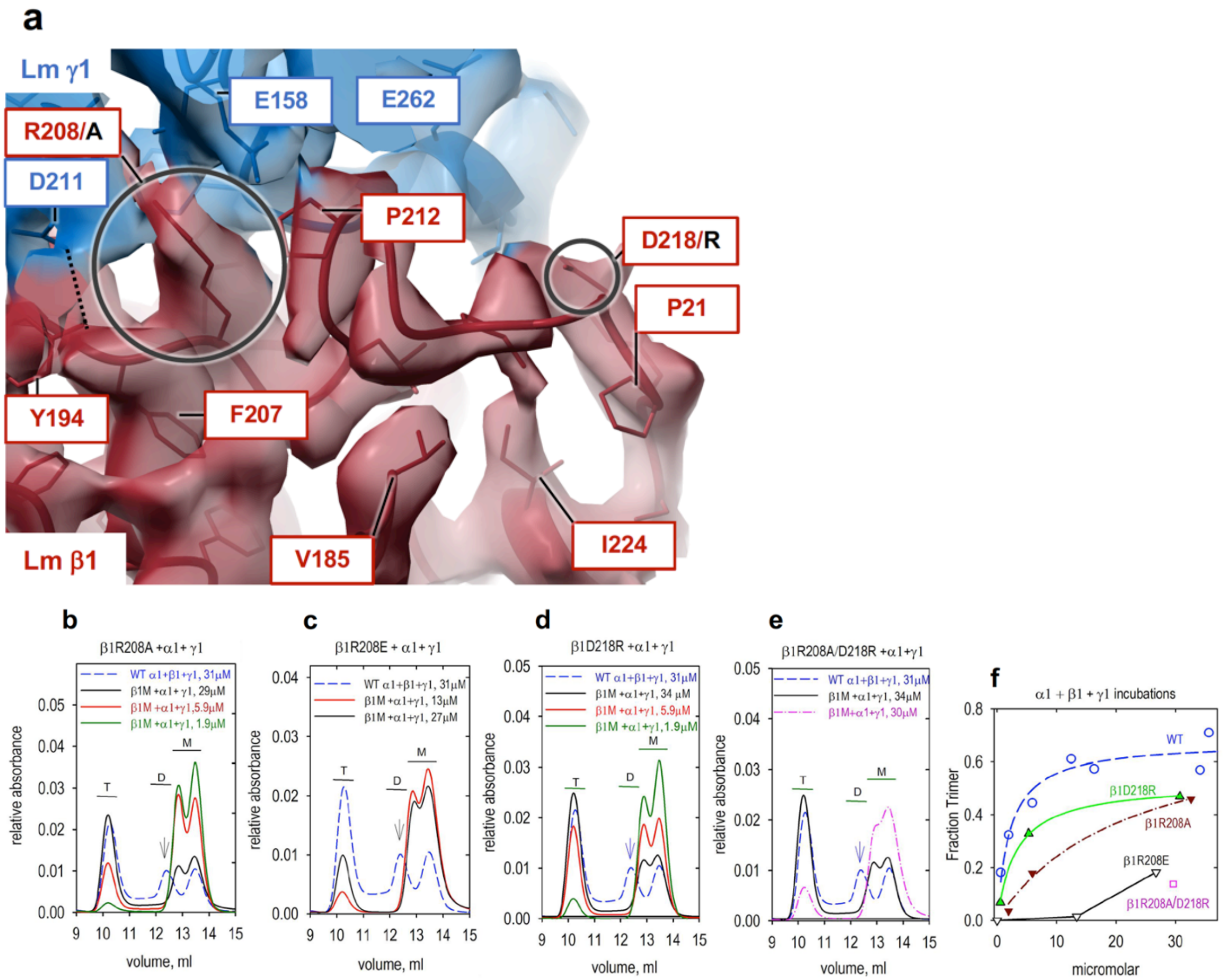

Supplementary Figure 13 Structural and functional analysis of the inter-subunit interfaces formed by β1 with γ1. The R208/A and R208/A-D218/R amino-acid substitutions were introduced to β1, analyzed by size-exclusion chromatography (SEC) and plotted as relative absorbances in comparison to previously reported R208/E and D218/R[4]. Based on the structural analysis, these mutations were predicted to affect the β1-γ1 binding interface. **(a)** R208 substitution with alanine was expected to destabilize the neighboring inter-subunit hydrogen bond involving Y194 from β1, and D211 from γ1, albeit less strongly than R208/E mutation (please see **Supplementary Figure 16c** for details). D218 is located at the inter-subunit interface with γ1, hence its substitution with arginine should also affect the β1-γ1 interaction. SEC profiles obtained for **(b)** R208/A **(c)** R208/E **(d)** D218/R, and **(f)** R208/A-D218/R show that amino-acid mutations described above disrupt the trimeric structure of the Lm polymer node as predicted. The trimeric (T), dimeric (D) and monomeric (M) protein fractions are labeled on the elution profiles. The SEC elution profiles obtained for a wild-type Lm polymer node are shown on the plots as blue dotted lines. **(f)** A graph showing a fraction of trimers vs. initial concentration of all components in the mixture. As predicted, R208/E has more severe effect on the formation of the trimeric Lm polymer node than R208/A, but less severe than a double β1 mutant (R208/A-D218/R). Source data are provided as a Source Data file.

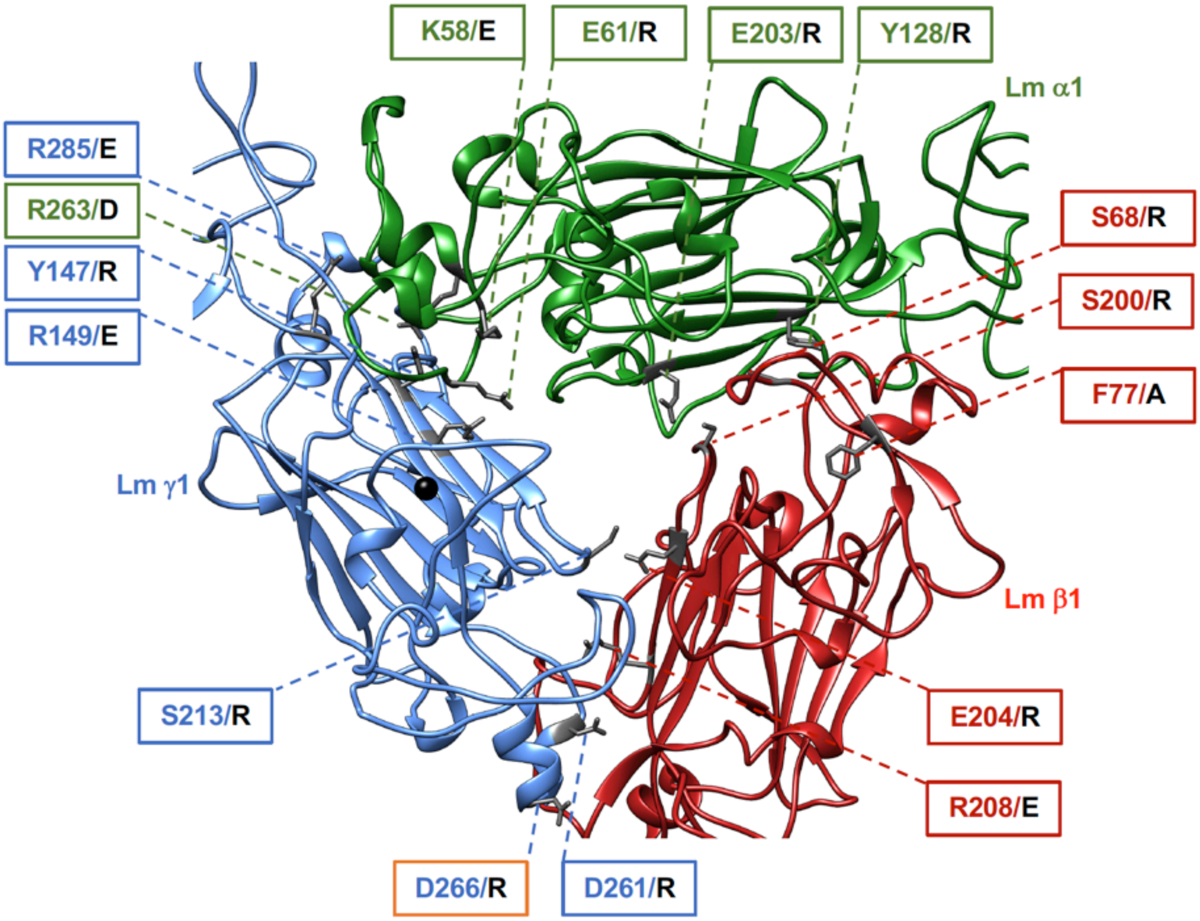

Supplementary Figure 14 The mutagenesis analysis of the trimeric Lm polymer node. Previously described amino acid substitutions[4], which resulted in disruption of Lm oligomeric structure can be mapped into inter-subunit interfaces in the cryo-EM structure of Lm polymer node. Mutations incorporated into α1, β1 and γ1 are highlighted in green, red and blue boxes, respectively. The γ1D266R mutation stabilizing the complex is located at the interface of γ1 and β1 subunits (orange box).

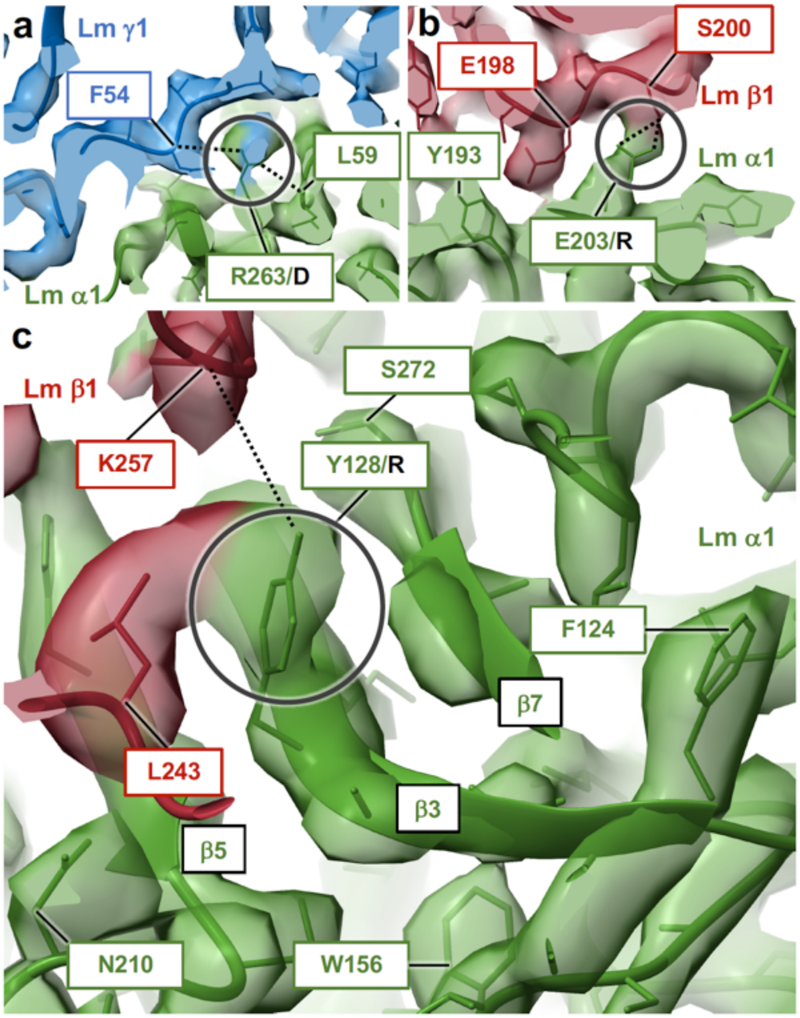

Supplementary Figure 15 Structural analysis of the inter-subunit interfaces formed by α1 with β1 and γ1 explains mechanistic basis for the amino-acid substitutions disrupting the trimeric structure of the Lm polymer node. Previous SEC analysis of the Lm polymer nodes reconstituted with the wild-type β1 and γ1, and a mutant α1 harboring one of the following single amino-acid substitutions: R263/D, E203/R and Y128/R, revealed that the altered Lm polymer nodes don't maintain their trimeric structures[4]. The cryo-EM structure shows that R263, E203 and Y128 are involved in stabilization of the inter-subunit interfaces within the trimeric Lm polymer node. **(a)** The substitution of R263 with aspartic acid in α1 breaks the hydrogen bonds formed by arginine with F54 from γ1 and L59 from α1, leading to disruption of the trimeric structure of the Lm polymer node. **(b)** A mutation of E203 from α1 to arginine at the α1-β1 interface disrupts two hydrogen bonds formed by E203 with S200 from β1. **(c)** Another substitution of Y128 from α1 at the α1-β1 interface breaks a hydrogen bond formed by Y128 with K257 from β1, and also affects the hydrophobic interaction between Y128 and L243 from β1, destabilizing the inter-subunit interface.

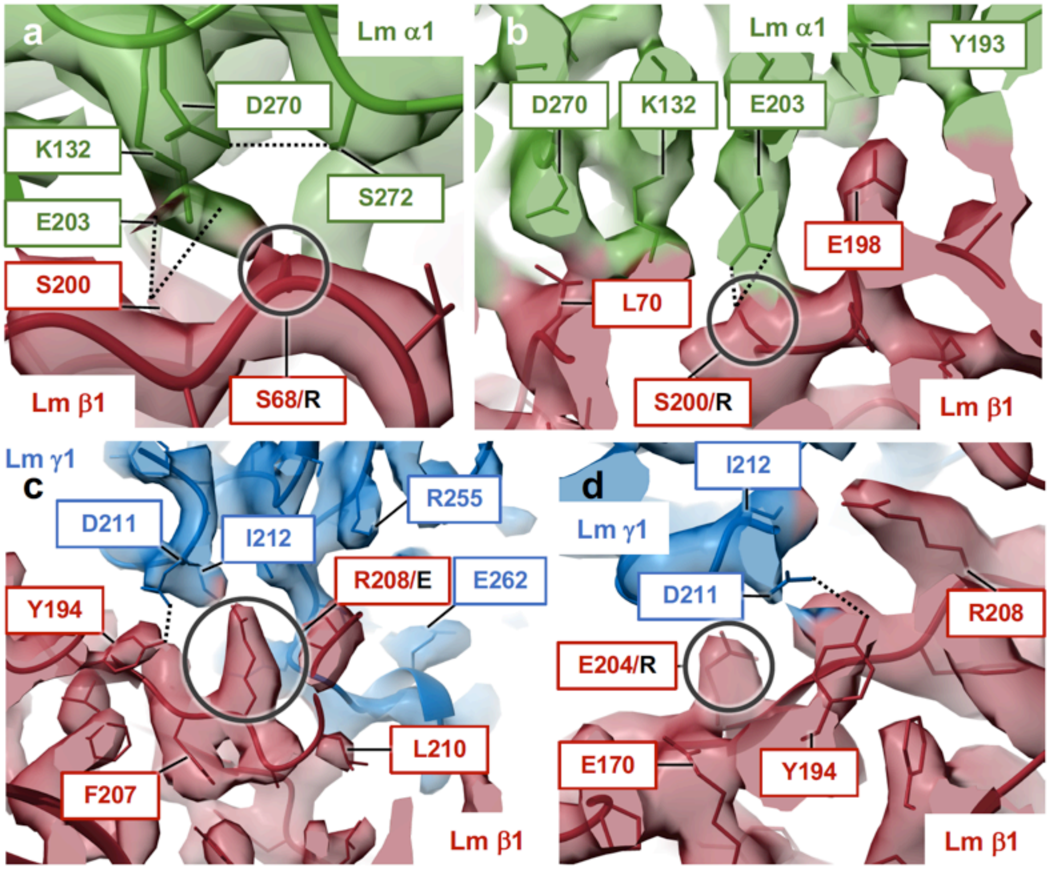

Supplementary Figure 16 Structural and functional analysis of the inter-subunit interfaces formed by β1 with α1 and γ1. Previous SEC analysis of the Lm polymer nodes reconstituted with the wild-type α1 and γ1, and a mutant β1 harboring one of the following single amino-acid substitutions: S68/R, S200/R, R208/E and E204/R, revealed that the altered Lm polymer nodes don't maintain their trimeric structures[4]. **(a)** The substitution of S68[5] with a positively-charged and bulky side-chain of arginine in β1 affects the neighboring network of hydrogen bonds stabilizing the β1-α1 interface, namely: a hydrogen bond between D270 and S272, both in α1, and most likely indirectly affects the inter-subunit hydrogen bond involving S200 in β1 and E203 in α1 through the electrostatic repealing of K132 from α1. The S68R mutation in a homologous Lm β2 has been shown to cause Pierson syndrome[6]. **(b)** A mutation of S200 to arginine in β1 disrupts the inter-subunit hydrogen bond formed by S200 from β1, and E203 from α1. **(c)** A substitution of R208 from β1 with a negatively charged side-chain of glutamic acid likely repeals D211 from γ1, affecting the hydrogen bond stabilizing the β1-γ1 interface, which involves Y194 from β1, and D211 from γ1. **(d)** The charge reversal from E204 to arginine in β1 most likely affects the neighboring hydrogen bond formed between D211 from γ1 and Y194 from β1. The arginine residue placed in this position likely forms the electrostatic interaction with a side chain of D211, which is involved in a hydrogen bond interaction.

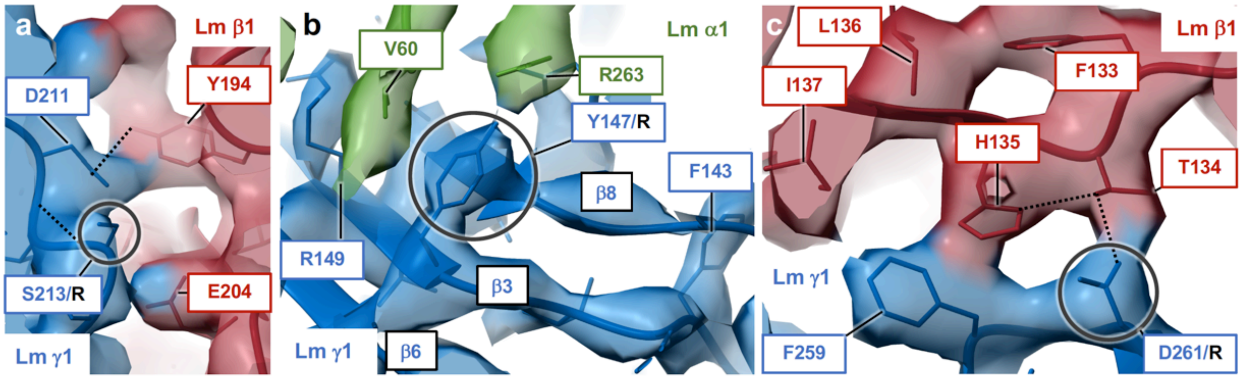

Supplementary Figure 17 Structural and functional analysis of the inter-subunit interfaces formed by γ1 with α1 and β1. Previous SEC analysis of the Lm polymer nodes reconstituted with the wild-type α1 and β1, and a mutant γ1 harboring one of the following single amino-acid substitutions: S213/R, Y147/R and D261/R, revealed that the altered Lm polymer nodes don't maintain their trimeric structures[4]. **(a)** Amino-acid substitution of S213 to arginine likely disrupts the hydrogen bond formed by D211 from γ1 and Y194 from β1. In addition, the inter-subunit hydrogen bond between S213 and D211 backbone may be disrupted, as well as the new electrostatic interaction of R213 and E204 from β1 might be formed, both affecting the γ1-β1 interface. **(b)** A substitution of Y147 from γ1 with a positively charged arginine disrupts the γ1-α1 interface by repealing the neighboring R263, and affecting the hydrophobic interaction of Y147 with V60, both residues located in α1 at the inter-subunit interface. **(c)** A mutation of D261 to arginine in γ1 disrupts the network of hydrogen bonds including the inter-subunit bond formed by D261 with T134 from β1, and intra-subunit hydrogen bond involving β1 residues T134 and H135.

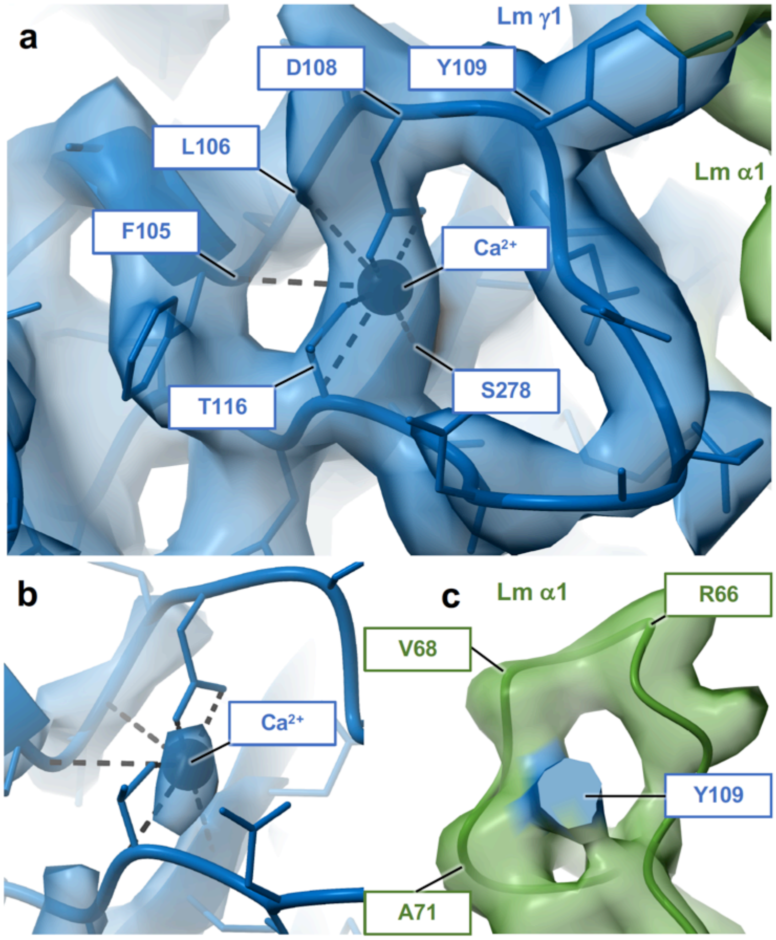

Supplementary Figure 18 Structural details of the α1-γ1 interface. (a) The loop connecting β-sheet strands β1 and β2 in γ1 forms the binding interface with α1. The loop also contains residues coordinating the calcium ion[7]. Lm γ1 residues involved in calcium coordination and some of the residues involved in binding to α1 are displayed in the figure. **(b)** A zoom into the calcium density in γ1. **(c)** The loop connecting the β-sheet strands β1 and β2 in α1 interacts with the analogous loop from γ1 shown above. The backbone protein density can be traced in this region, but the side chains are less well-defined. Some of the residues from the loop are labeled in the figure.

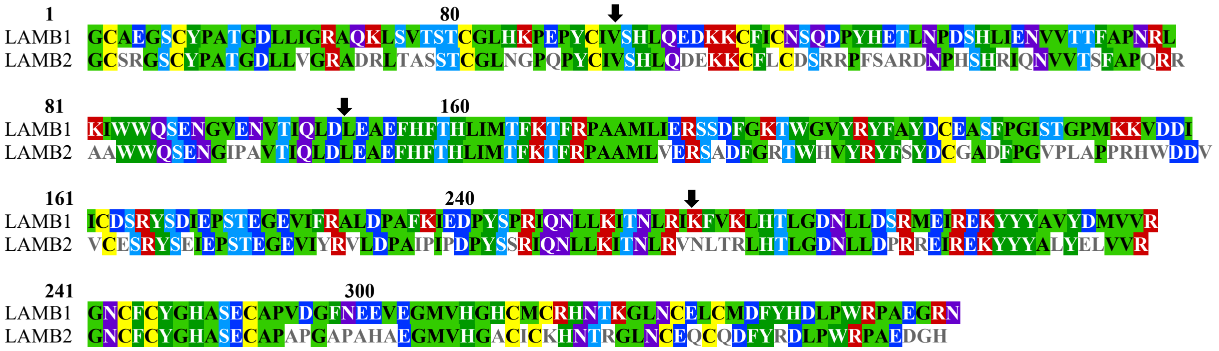

Supplementary Figure 19 Sequence alignment of Lm β1 and Lm β2. The alignment was performed in Clustal Omega 1.2.4. Both proteins share 72% sequence identity in LN domains. Residues implicated in Pierson syndrome are conserved in both Lm variants. These residues are indicated by black arrows.

Supplementary References

1. Pintilie, G. et al. Measurement of atom resolvability in cryo-EM maps with Q-scores. *Nat. Methods* **17**, 328-334 (2020).
2. Ramirez-Aportela, E., Mota, J., Conesa, P., Carazo, J.M. & Sorzano, C.O.S. DeepRes: a new deep-learning- and aspect-based local resolution method for electron-microscopy maps. *IUCrJ.* **6**, 1054-1063 (2019).
3. Jumper, J. *et al.* Highly accurate protein structure prediction with AlphaFold. *Nature* **596**, 583-589 (2021).
4. McKee, K.K., Hohenester, E., Aleksandrova, M. & Yurchenco, P.D. Organization of the laminin polymer node. *Matrix Biol.* **98**, 49-63 (2021).
5. Purvis, A. & Hohenester, E. Laminin network formation studied by reconstitution of ternary nodes in solution. *J. Biol. Chem.* **287**, 44270-7 (2012).
6. Matejas, V. *et al.* Mutations in the human laminin beta2 (LAMB2) gene and the associated phenotypic spectrum. *Hum. Mutat.* **31**, 992-1002 (2010).
7. Carafoli, F., Hussain, S.A. & Hohenester, E. Crystal structures of the network-forming short-arm tips of the laminin beta1 and gamma1 chains. *PLoS One* **7**, e42473 (2012).
