# Supplementary material for: Cryo-EM reveals the molecular basis of laminin polymerization and LN-lamininopathies
Source: Nat Commun. 2023 Jan 19;14:317. doi: 10.1038/s41467-023-36077-z (PMC9852560; doi:10.1038/s41467-023-36077-z)
Supplement: Supplementary file 3 — Description of additional Supplementary File [file 41467_2023_36077_MOESM3_ESM.pdf]

<u>Descriptions of Additional Supplementary files</u>

Supplementary Movie 1: Conformational changes in the structure of the Lm polymer node. Individual subunits are colorcoded i.e. a1, b1 and g1 are shown in green, red and blue, respectively. The LE rods from all three Lm subunits display the rotational motion along the long axes, along with the planar swinging of the LE domains along the axes perpendicular to these long axes. The analysis was performed with an unrefined set of 213,693 particle images obtained after the initial 3D hetero-refinement, which removed artifacts and Lm monomers from the data-set, but prior to subsequent extensive structural refinements
