# Supplementary material for: Cryo-EM reveals the molecular basis of laminin polymerization and LN-lamininopathies
Source: Nat Commun. 2023 Jan 19;14:317. doi: 10.1038/s41467-023-36077-z (PMC9852560; doi:10.1038/s41467-023-36077-z)
Supplement: Supplementary file 5 — Reporting Summary [file 41467_2023_36077_MOESM5_ESM.pdf]

nature portfolio

Corresponding author(s): Arek Kulczyk

Last updated by author(s): Dec 30, 2022

Reporting Summary

Nature Portfolio wishes to improve the reproducibility of the work that we publish. This form provides structure for consistency and transparency in reporting. For further information on Nature Portfolio policies, see our Editorial Policies and the Editorial Policy Checklist.

Statistics

For all statistical analyses, confirm that the following items are present in the figure legend, table legend, main text, or Methods section.

n/a	Confirmed	
☐	☒	The exact sample size (*n*) for each experimental group/condition, given as a discrete number and unit of measurement
☐	☒	A statement on whether measurements were taken from distinct samples or whether the same sample was measured repeatedly
☒	☐	The statistical test(s) used AND whether they are one- or two-sided<br>*Only common tests should be described solely by name; describe more complex techniques in the Methods section.*
☐	☒	A description of all covariates tested
☐	☒	A description of any assumptions or corrections, such as tests of normality and adjustment for multiple comparisons
☐	☒	A full description of the statistical parameters including central tendency (e.g. means) or other basic estimates (e.g. regression coefficient) AND variation (e.g. standard deviation) or associated estimates of uncertainty (e.g. confidence intervals)
☒	☐	For null hypothesis testing, the test statistic (e.g. F, t, r) with confidence intervals, effect sizes, degrees of freedom and P value noted<br>*Give P values as exact values whenever suitable.*
☐	☒	For Bayesian analysis, information on the choice of priors and Markov chain Monte Carlo settings
☐	☒	For hierarchical and complex designs, identification of the appropriate level for tests and full reporting of outcomes
☒	☐	Estimates of effect sizes (e.g. Cohen's *d*, Pearson's *r*), indicating how they were calculated

Our web collection on statistics for biologists contains articles on many of the points above.

Software and code

Policy information about availability of computer code

Data collection	EPU 2, SerialEM 4.0
Data analysis	AlphaFold2, Clustal Omega 1.2.4, COOT 0.9, cryoSPARC v3, Molprobity 4.02, Phenix 1.18, PyEM, Relion-3, Scipion-3, UCSF Chimera, UCSF ChimeraX, Waters_connect

For manuscripts utilizing custom algorithms or software that are central to the research but not yet described in published literature, software must be made available to editors and reviewers. We strongly encourage code deposition in a community repository (e.g. GitHub). See the Nature Portfolio guidelines for submitting code & software for further information.

Data

Policy information about availability of data

All manuscripts must include a data availability statement. This statement should provide the following information, where applicable:

- Accession codes, unique identifiers, or web links for publicly available datasets
- A description of any restrictions on data availability
- For clinical datasets or third party data, please ensure that the statement adheres to our policy

Atomic coordinates of Lm polymer node generated in this study were deposited in the Protein Data Bank (PDB) under accession code 8DMK. The sharpened and unsharpened cryo-EM maps were deposited in the Electron Microscopy Data Bank (EMDB) under accession code EMD-27542. In addition, we used in this study atomic coordinates files with the following accession codes: 2Y38, 4AQS, 4AQT.

Human research participants

Policy information about studies involving human research participants and Sex and Gender in Research.

Reporting on sex and gender	The project did not involve human research participants.
Population characteristics	The project did not involve human research participants.
Recruitment	The project did not involve human research participants.
Ethics oversight	The project did not involve human research participants.

Note that full information on the approval of the study protocol must also be provided in the manuscript.

Field-specific reporting

Please select the one below that is the best fit for your research. If you are not sure, read the appropriate sections before making your selection.

☒ Life sciences ☐ Behavioural & social sciences ☐ Ecological, evolutionary & environmental sciences

For a reference copy of the document with all sections, see nature.com/documents/nr-reporting-summary-flat.pdf

Life sciences study design

All studies must disclose on these points even when the disclosure is negative.

Sample size	We acquired a total of 44743 movies with 300 kV Titan Krios/K3. Particle selection and 2D classification procedures generated a set of 1,068,172 particle images used for structure calculations. Due to the intrinsic dynamics and heterogeneity in the sample, the quality/ resolution of the reconstructions didn't improve by including additional particles images. The final structure was calculated with a set of 125,011 particle images. Reducing this set of particles further negatively affected the quality/resolution of the reconstructions.
Data exclusions	No data were excluded from the analysis.
Replication	Prior to final data collections, we extensively screened samples. We discovered and carefully characterized time- and concentration-dependent instability, and the orientation bias of Lm trimers. This information, in turn, allowed us to develop a highly reproducible sample preparation scheme for final data collections. The reproducibility was confirmed in a total of sixteen multi-day data collection sessions, and consistent results of subsequent 2D and 3D analyses.
Randomization	Particle images were automatically selected from averaged micrographs. 2D templates and the initial model were derived from within the data sets. The resolution of the final structure was calculated according to the gold-standard FSC.
Blinding	All data acquisition and processing steps were performed in an unbiased and automated manner according to the current standards in the field of cryo-EM.

Reporting for specific materials, systems and methods

We require information from authors about some types of materials, experimental systems and methods used in many studies. Here, indicate whether each material, system or method listed is relevant to your study. If you are not sure if a list item applies to your research, read the appropriate section before selecting a response.

Materials & experimental systems

n/a	Involved in the study	
☒	☐	Antibodies
☐	☒	Eukaryotic cell lines
☒	☐	Palaeontology and archaeology
☒	☐	Animals and other organisms
☒	☐	Clinical data
☒	☐	Dual use research of concern

Methods

n/a	Involved in the study	
☒	☐	ChIP-seq
☒	☐	Flow cytometry
☒	☐	MRI-based neuroimaging

Eukaryotic cell lines

Policy information about cell lines and Sex and Gender in Research

Cell line source(s)	Human embryonic kidney cell line HEK293 obtained from ATCC (CRL-1573).
Authentication	HEK293 cells were authenticated by morphology and growth characteristics, the absence of laminin expression, and transfectability to generate secreted tagged laminin glycoprotein products.
Mycoplasma contamination	The cells tested negative for mycoplasma contamination.
Commonly misidentified lines (See ICLAC register)	None. Commercially available HEK293 cells were authenticated by ATCC, and cultivated in the presence of appropriate selection markers (i.e. puromycin, zeocin or G418).
